# Supplementary material for: Early Surgery with Neuraxial Anaesthesia in Patients on Chronic Antiplatelet Therapy with a Proximal Femur Fracture: Multicentric Randomised Clinical Trial
Source: J Clin Med. 2021 Nov 18;10(22):5371. doi: 10.3390/jcm10225371 (PMC8624584; doi:10.3390/jcm10225371)
Supplement: Supplementary file 1 [file jcm-10-05371-s001.zip › jcm-1456768-supplementary.pdf]

Journal of
Clinical Medicine

MDPI

Table S1. Effect of the early platelet function-guided surgery strategy on the admission-to-surgery interval: crude regression coefficients.

	Bivariate model				
	**Mean time to surgery in delayed surgery** (b0)	**Early guided surgery** (b1)	**Co-variable** b2	p	**Model R2** (%)
Intervention group	5.2	−2.4	-	-	29.5
Age (per year)	4.2	−2.4	0.0	0.56	29.7
≥80 years	4.9	−2.4	0.4	0.91	29.9
Sex (male)	5.0	−2.4	0.5	0.17	30.5
ASA score	3.3	−2.5	0.6	0.12	30.7
4 (vs ≤3)	5.15	−2.43	1.0	0.01	31.3
Comorbidities					
Arterial hypertension	4.9	−2.45	0.4	0.38	29.9
Surgery	5.5	−2.39	−0.4	0.22	30.3
Ischemic neurological disease	5.2	−2.46	−0.04	0.90	29.5
Delirium	5.4	−2.52	−0.6	0.08	31.1
Diabetes	5.2	−2.46	0.6	0.90	29.5
Coronary disease	5.2	−2.48	0.2	0.49	29.8
Chronic renal insufficiency	5.1	−2.47	0.3	0.43	29.8
Pulmonary disease					
Oncological disease	5.3	−2.45	−0.4	0.34	30.0
Atrial fibrillation	5.2	−2.45	0.2	0.62	29.6
	5.2	−2.48	0.6	0.23	30.2
Antiplatelet drug					
P2Y12-RI	4.6	−2.4	0.7	0.01	30.9
Orthopaedic treatment					
Osteosynthesis					
	5.2	−2.46	0.01	0.967	29.5
Type of anaesthesia					
General +/- peripheral nerve block	5.2	−2.45	0.10	0.848	29.5
Hospitals					
Hospital 1	4.5	−2.4	1.4	<0.001	38.6
Hospital 2	5.4	−2.4	−1.1	<0.01	33.3
Hospital 3	5.4	−2.5	−1.1	<0.01	33.4
Hospital 4	5.2	−2.4	0.2	0.67	29.6

The table shows the mean admission-to-surgery interval for the control group (b0), the effects of this interval on the early surgery group (b1, negative, because time is saved), and the effects of different covariates listed on the left (b2). In each case, the effect of increasing each b2 can be seen in b0 and b1. ANOVA approximation for random effect of hospitals show a constant reduction of 2.3 days ($p<.001$) on the early surgery group, with no clustering effect. Outliers were removed to improve the linear regression model fit. Data outside the IQR change the Pearson correlation coefficient from 0.551 to 0.771 and change the effect of the experimental group from 2.7 to 2.4 days. A total of 7 outliers were removed (3 in the experimental group and 4 in the control group).

Table S2. Adverse events (AEs) per study arm for 30±15 days post-discharge

	**Early surgery** ***n*** **(%)**	**Delayed surgery** ***n*** **(%)**
All AEs	23	24
Resolved	14 (60.87)	15 (62.50)
AE classification		
Moderate	8 (34.78)	8 (33.33)
Serious	15 (65.22)	16 (66.67)
Serious ending in death	9 (39.13)	9 (37.50)
Number of patients with AEs:	21	19
Age: mean (SD)	85.14 (6.7)	84.81 (11.9)
Women	10 (47.62)	10 (52.63)
